# Inflammation across the spectrum of cardiovascular disease: healthcare physician perceptions and practices in six European countries

**DOI:** 10.3389/fcvm.2026.1672079

**Published:** 2026-09-25

**Authors:** Guiomar Mendieta, Morten Schou, Jelena Vukmirica, Henna Cederberg-Tamminen, Edmundo Erazo-Tapia, Roman Casciano, Stephanie Read, Jelena Sostar, Marine Mathias, April Dominique Ocampo, Andrea Bernardini, Jan H. Cornel

**Affiliations:** 1 Department of Cardiology, Hospital de la Santa Creu I Sant Pau, Barcelona, Spain; 2 Centro Nacional de Investigaciones Cardiovasculares (CNIC), Madrid, Spain; 3 Department of Cardiology, Copenhagen University Hospital–Herlev & Gentofte, Copenhagen, Denmark; 4 Novo Nordisk A/S, Copenhagen, Denmark; 5 Certara, New York, NY, United States; 6 Certara, London, United Kingdom; 7 Certara, Milan, Italy; 8 Certara, Paris, France; 9 Certara, Makati City, Philippines; 10 Department of Cardiology, Radboud University Medical Center, Nijmegen, Netherlands; 11 Department of Cardiology, Northwest Clinics, Alkmaar, Netherlands

**Keywords:** atherosclerotic cardiovascular disease, chronic kidney disease, heart failure, hsCRP (high-sensitivity c-reactive protein), systemic inflammation

## Abstract

**Background:**

There is growing evidence that inflammation is associated with development and progression of cardiovascular disease (CVD). The extent of physician awareness of this evidence, and its impact on clinical care, is unknown. Therefore, our aim was to investigate and characterize the perceptions and clinical practices of HCPs with respect to inflammation when treating patients with atherosclerotic CVD (ASCVD) + chronic kidney disease (CKD) stage 3–4 or heart failure (HF).

**Methods:**

This was a qualitative research study involving semi-structured, 1-hour Zoom interviews (including open- and close-ended questions) conducted from February-April 2024. Eligible HCPs were cardiologists, nephrologists, or general practitioners with ≥5 and ≤30 years of post-graduate clinical experience; working in France, Denmark, the Netherlands, Spain, Sweden, and Switzerland; who had managed ≥10 patients with ASCVD + CKD or HF in the previous 3 months.

**Results:**

Most of the 62 HCPs included in the study (84%) acknowledged the theoretical importance of inflammation in progression of ASCVD + CKD or HF. However, only 17 (27% of the 62) routinely tested for high-sensitivity C-reactive protein (hsCRP) due to limited awareness of the clinical implications of inflammation in CVD and the lack of consistently proven interventions. If hsCRP is assessed, HCPs cannot alter their patient management in response to the findings due to a lack of treatment options.

**Conclusion:**

This study presents the perceptions and practices of a sample of HCPs. Because of limited evidence for the efficacy of interventions and the incomplete understanding of the role of inflammation in CVD, inflammation remains a neglected treatment target in clinical practice.

## Introduction

Cardiovascular disease (CVD) is still the leading cause of death globally (1), despite the fact that the incidence of and mortality from CVD [including atherosclerotic cardiovascular disease (ASCVD) and heart failure (HF)] have declined over the past several decades (2, 3). Improved treatments and decreased prevalence of traditional risk factors (e.g., smoking, high blood pressure, diabetes, and hyperlipidemia), together with earlier detection of disease through improved cardiac biomarkers, have contributed to this decline (2). However, CVD continues to impose a substantial health and economic burden (1).

Inflammation (formerly, systemic inflammation) has been demonstrated to affect the initiation, development, and progression of atherosclerotic plaques in ASCVD (4–7). Inflammation has also been suggested to play a role in HF (8) including HF with preserved ejection fraction (HFpEF) (2, 9). The prevalence of inflammation in CVD is rising (10) due to increased obesity (11), type 2 diabetes (12), and chronic kidney disease (CKD) (13) worldwide. While there is growing evidence that inflammation plays a role in ASCVD development and progression, the perceptions and practices of Healthcare physicians (HCPs) regarding inflammation in CVD is limited. In the cross-sectional FLAME-ASCVD survey-based study, 71% of eligible cardiologists from 10 countries indicated that systemic inflammation was a risk factor for ASCVD (14, 15). In the FLAME-ASCVD-Nephro survey, though nephrologists considered systemic inflammation a CV risk factor among ASCVD + CKD patients, they were more likely to discuss traditional CV risk factors with patients (15). Systemic inflammation was only the ninth most common ASCVD risk factor nephrologists discussed with their patients (15).

Elevated inflammatory biomarkers, such as C-reactive protein (CRP) and interleukin (IL)-6, have been shown to be associated with higher risk for development of CVD events in apparently healthy men (16) and women initially free of CVD (17); with development of high blood pressure or hypertension (18) and increased risk of death or major adverse cardiovascular events (MACE) in patients with a history of myocardial infarction (MI) in secondary care settings (19).

Findings from the CANTOS trial (Canakinumab Anti-Inflammatory Thrombosis Outcomes Study; NCT01327846) suggest that targeting inflammation can improve CVD outcomes. Specifically, the trial demonstrated that inhibition of IL-1β, a cytokine central to the inflammatory response, with canakinumab, reduced the rate of recurrent cardiovascular events, independent of blood pressure or cholesterol reduction, among patients with a prior MI (6, 20). Secondary analyses of the CANTOS trial demonstrated greater CV risk reductions with greater on-treatment reductions in CRP and IL-6 (21, 22). Similar findings were observed among patients with stage 3 CKD, a patient group at higher risk of ASCVD, but with few effective risk-reducing therapies available (21, 23). In addition, evidence from the phase 2 RESCUE trial (Trial to Evaluate Reduction in Inflammation in Patients With Advanced Chronic Renal Disease Utilizing Antibody Mediated IL-6 Inhibition; NCT03926117) showed that direct inhibition of IL-6 with ziltivekimab markedly reduced high-sensitivity (hs) CRP and thrombosis biomarkers in a dose-dependent manner among patients with stage 3–5 CKD and elevated hsCRP (5).

The CANTOS trial also demonstrated that baseline levels of IL-6 and CRP can predict future hospitalization for HF in patients with prior MI, although left ventricular ejection fraction was not captured in the study (24). In addition, recent studies suggest that inflammation in patients with HFpEF increases the risk of both CV and all-cause death (25) and that patients with HFpEF may benefit from anti-inflammatory therapies based on the increased level of inflammation and increased risk associated with high levels of inflammation in these patients (26).

Randomized double-blind clinical trials have also demonstrated the efficacy of low-dose colchicine, an oral anti-inflammatory medication that reduces CRP as measured by hsCRP testing in secondary prevention in patients with CVD (27). In patients with a recent MI, there was a significantly lower risk of CV events with colchicine treatment (28). Additionally, low-dose colchicine was shown to reduce the risk of CV events in patients with chronic coronary disease (29).

Several other drugs with varying primary mechanisms of action that also have an effect on inflammation have been shown to be beneficial in patients with CVD. These include lipid-lowering therapies (30), sodium glucose cotransporter-2 inhibitors (SGLT2i) (31, 32), glucagon-like peptide-1 receptor agonist (GLP-1RA) (33), and the non-steroidal mineralocorticoid receptor antagonist, finerenone (34).

Clinical guidelines at the time that this study was conducted offered little in terms of recommendations for assessing and managing inflammation in patients with ASCVD, advanced CKD (stage 3–4), or HF (35–37). However, since 2021, the European Society of Cardiology (ESC) has stated that low-dose (27), may be used for secondary prevention if other risk factors are not sufficiently controlled or if CVD events recur (36). More recent ESC 2024 guidelines, updated after the conduct of this study, recommend that low-dose colchicine “should be considered to reduce MI, stroke, and need for revascularization” in patients with coronary artery disease and that hsCRP testing should be considered in the initial diagnosis of individuals with suspected chronic coronary syndrome (both Class IIa recommendations) (38).

Inflammation has been shown to play a role in the initiation and progression of CVD. However, it remains unclear how this evidence is currently being applied in routine clinical practice. The importance of inflammation in CVD on risk assessment, treatment, and clinical management practices by (HCPs) that treat patients with CVD is unknown. Therefore, the objective of this qualitative research study (Study ID: NN6018-7883) was to explore and characterize the perceptions and clinical management practices of HCPs in 6 European countries treating patients across the spectrum of CVD, including ASCVD + CKD stage 3–4 or HF, using semi-structured interviews to better understand the current role of inflammation in the clinical care pathways for CVD.

## Methods

### Study design

This was a qualitative research study that involved semi-structured, double-blinded, 1-hour, in-depth interviews with HCPs conducted between February and April 2024. Participants were recruited from the proprietary M3 Global database of pre-screened panellists (M3 Global Research, Fort Washington, PA, USA). Participants were invited to an online screening survey. Those who qualified were scheduled, rescreened, and confirmed to be interviewed. Interviews were conducted via Zoom, and interview data were stored on secure servers. Screeners had ≥5 years of experience in market research and listed cardiology as an area of specialty. A thematic analysis was applied using the framework method. Themes were predefined based on the study objectives and existing literature. Interview content was systematically extracted and compared across these themes and across specialties. For the purposes of this study, patients with ASCVD were defined as those with a history of acute coronary syndrome (MI and unstable angina), atherosclerotic stroke, carotid stenosis, stable coronary heart disease, atherosclerotic transient ischemic attack, or lower extremity artery disease. Patients with CKD stage 3–4 were defined as those with a glomerular filtration rate of ≥15 and <60 mL/min/1.73m^2^ (39–41). HF was defined as HFpEF [≥50% left ventricular ejection fraction (LVEF)] or HF with mid-range EF (HFmrEF) (41% to 49% LVEF). HFpEF and HFmrEF were the focus of this study given the ongoing evaluation of anti-inflammatory treatments, including Il-1 and Il-6 blockers, for these types of HF (42, 43).

### Setting and study population

HCPs were recruited from France, Denmark, the Netherlands, Spain, Sweden, and Switzerland. Purposive sampling was used to capture information from each specialty. A pre-specified recruitment target of 62 HCPs was applied. The target included 32 HCPs managing patients with ASCVD + CKD and 30 HCPs managing patients with HF (Supplementary Table S1).

Eligible HCPs were cardiologists, nephrologists, or general practitioners (GPs) with post-graduate clinical experience from 5 to 30 years. In addition, eligible HCPs had managed at least 10 patients with either ASCVD + CKD or HF in the previous 3 months. This requirement was applied to ensure respondents had meaningful clinical exposure to cardiovascular disease management. HCP's with >30 years of experience were excluded to capture the physician population with a relevant role in managing both today's patient population as well as the future patient population.

This study consisted of non-interventional qualitative interviews with healthcare professionals regarding their clinical perspectives on inflammation in cardiovascular disease. No patients or patient data were involved. Informed consent was obtained, and data were anonymized and analyzed in aggregate. As the study involved only expert opinion research with no patient data or intervention, formal ethics committee/IRB review was not required.

### Data collection

Data were collected via 1-hour semi-structured interviews using a comprehensive discussion guide including both open- and close-ended questions. The interviews were conducted by a single interviewer per country from February to April 2024. The interviewers were all market research professionals with 5 to <30 years of experience and each interviewer cited cardiology as an area of expertise. All HCP interviews were audio recorded and transcribed verbatim. The study used a total of 3 French, 4 Spanish, and 2 Nordic (one for each language) transcribers. The discussion guide was piloted among 3 eligible HCPs prior to data collection and adapted accordingly. The discussion was structured around 5 key areas of the clinical care pathway including (1) risk assessment and early detection, (2) diagnosis and referral to a specialist, (3) initial treatment, (4) ongoing management, and (5) rehabilitation and secondary prevention.

The interviews began with a discussion around clinical pathways, without specific probes into systemic inflammation, to allow HCPs to bring up inflammation unprompted. To understand physician's baseline level of awareness and consideration relating to the role of inflammation in CVD risk, a 2-stage interview process was applied. First, we provided physicians with the opportunity to bring up inflammation unprompted to understand whether inflammation is a routine consideration in their clinical practice. If the HCPs did not bring up inflammation unprompted, the interviewer raised the topic and asked specific questions. Once inflammation was raised by an interviewee during the call, a specific series of further questions on the topic were asked. If inflammation was not mentioned by HCPs unprompted, a different series of questions were asked. Following discussion of the general clinical care pathways, the topic of inflammation was raised by interviewers and questions specific to systemic inflammation were asked in a structured manner covering all phases of the clinical pathway. Not all questions were posed to all interviewees. Some questions asked were dictated by the response to the previous question. Although the sample size was predefined based on the need to cover relevant expert roles, the depth of the discussion guide and the consistency of insights across interviews indicated that thematic saturation was achieved.

The discussion guide covered the following themes based on study objectives and existing literature. The first theme covered HCP perceptions of the role of inflammation in ASCVD + CKD or HF, separately. Specific topics included the role of inflammation in development, progression, and risk for CV events. The second theme covered the effect of inflammation on current clinical care pathways for these patient populations. Specific topics included the assessment/detection of inflammation, including the use of hsCRP testing, the impact of inflammation on treatment and management practices including the use of colchicine, and barriers to the assessment and management of inflammation in these patient populations. This study focused specifically on hsCRP as a key biomarker since the International Atherosclerosis Society has identified this as the most consistent and reliable marker of CV risk and hsCRP is also a key biomarker in ongoing clinical trials (44).

The Discussion Guide was designed to capture the attitudes and practices of the physicians relating to the following themes:

- Current clinical care pathways focusing on specific pathway trigger points (risk assessment & early detection, diagnosis & referral to specialist, initial treatment, ongoing management, rehabilitation & secondary prevention) for:
  - ASCVD with CKD stage 3–4 in general and systemic inflammation
  - HF in general and systemic inflammation
- The role of systemic inflammation in development, progression and as a risk factor for CVD events in patients with ASCVD + CKD stage 3–4 or HF
- The impact of systemic inflammation on current as well as future clinical care pathways
- The measurement of hsCRP, including timing, frequency of testing, patient population, and by specific physician specialty
- The impact of clinical guidelines on the management of systemic inflammation
- Barriers and gaps in the current clinical pathway that hinder the delivery of optimal patient care
- Perceived requirements for establishing systemic inflammation as an independent risk factor being routinely measured and treated by HCPs

### Data analysis

Interviews were transcribed in their original language and translated into English by qualified native-language translators and/or verified native speakers, ensuring the accurate preservation of contextual meaning.

English-language transcripts were independently reviewed by at least two members of the research team. Responses were extracted into a structured Microsoft Excel database and coded according to predefined thematic areas and study-specific scoring criteria. Coding was reviewed by multiple researchers, with discrepancies resolved through discussion and consensus, re-examining the source transcript and translation where necessary.

Findings are reported overall and by specialty. Descriptive statistics (numbers and percentages) were used to summarize structured questions with binary or categorical answers where appropriate. Key themes were extracted from interview transcripts and germane quotations from HCP interviews are presented.

## Results

### Study population

A total of 1090 HCPs were sent a screening document prior to determining their eligibility for this study (Supplementary Table S2). This resulted in 168 replies, and 101 HCPs were deemed eligible for inclusion (Supplementary Table S3). The 101 eligible HCPs were prioritized for interviews based on their specialty to fill the quota from each country, years of practice, and number of patients treated. A total of 62 HCPs were included in the study: 10 HCPs each from Denmark, Sweden, the Netherlands, and Switzerland and 11 HCPs each from France and Spain. The study population included 36 cardiologists, 12 nephrologists, and 14 GPs (Table 1). Included HCPs had a mean (range) post-graduate clinical experience of 16.2 years (6–29). In the 3 months prior to the interview, cardiologists had consulted with a mean (range) of 279 (30–800) ASCVD patients, 78 (10–300) ASCVD + CKD patients, and 252 (30–1200) HF patients. Nephrologists had consulted with 193 (20–650) CKD patients. GPs had consulted with 272 (25–1500) ASCVD patients, 121 (12–700) CKD stage 3–4 patients, 83 (5–300) ASCVD + CKD stage 3–4 patients, and 109 (15–300) HF patients. Due to difficulty in recruiting GPs meeting the ≥10 patient criterion, an exception to the eligibility criteria was made for GPs, allowing inclusion if they managed ≥5 ASCVD + CKD patients in the past 3 months. HCP quotations relating to their perceptions of inflammation in CVD are reported in Table 2.

**Table 1:** Respondent characteristics (*N* = 62).

Characteristics	Cardiologists *N* = 36	Nephrologists *N* = 12	GP *N* = 14
Length of practice, mean (range)	15 (6–28)	16 (7–29)	17 (6–35)
Patients seen in the last 3 months, average (range)			
ASCVD	279 (30–800)	N/A	272 (25–1,500)
CKD stage 3–4	N/A	193 (20–650)	121 (12–700)
ASCVD + CKD stage 3–4	78 (10–300)	N/A	83 (5–300)
HF	252 (30–1,200)	N/A	109 (15–300)

ASCVD, atherosclerotic cardiovascular disease; CKD, chronic kidney disease; CV, cardiovascular; GP, general practitioner; HF, heart failure; N/A, not applicable.

**Table 2:** HCP interview quotations.

Specialty	Years in practice	Quotation
**Systemic inflammation as an independent CV risk factor**		
General cardiologist	25	“Right now, though, there's not much we can do in terms of inflammation, except referring them to the rheumatologist, if you suspect that there's a rheumatological disease behind it.”
General cardiologist	15	“However, systemic inflammation is a modifier factor. I know that a patient with systemic inflammatory disease of any nature, or with rheumatoid arthritis, who undergoes a therapy has a greater risk of kidney function impairment, has a greater risk of ischemic heart disease, has a worse evolution despite re-vascularization, has a greater risk of associated diabetes. The prognosis of these patients is worse. Systemic inflammation is not a conventional risk factor but a modifying factor or a factor that worsen the disease.”
General practitioner	25	“I do know that there are many indicators which prove that inflammation does play a role in the development of heart failure. Because I might not be sufficiently well informed, quite simply, to give an answer which is really open and definitive. I am not really, how should I put this? Sufficiently specialized in cardiology to answer these questions.”
Nephrologist	29	“Yes, I can agree [that systemic inflammation is an independent CV risk factor] because it can speed up progression of atherosclerosis.”
Nephrologist	8	“I would assume [systemic inflammation is an independent CV risk factor]. Because it affects and destroys the vascular system.”
General practitioner	14	“[Systemic inflammation is an independent CV risk factor] because we know that it leads to inflammation in the arterial vessel and therefore promotes cardiovascular events.”
**Systemic inflammation in development and progression of ASCVD**		
General cardiologist	15	“It has an important role. Independently, taken in isolation of all the rest, we have difficulty interpreting it. However, in a patient who already has one or more cardiovascular risk factors, the inflammatory marker test makes sense, because it highlights, it corroborates the fact that the patients are at risk of presenting a major cardiovascular event over the coming years.”
**Systemic inflammation in development and progression of CKD**		
Nephrologist	28	“..we know that patients that have active inflammation have more rapid kidney function loss. At the same time, they also have more CV events, there is evidence of that.”
**Systemic inflammation caused by autoimmune diseases**		
General cardiologist	12	“Autoimmune diseases, rheumatological diseases, diseases with chronic inflammation. Several studies claim that vascular risk increases if severe-extensive psoriasis is not treated. Everything goes in the same line. Vascular inflammation or systemic inflammation increases cardiovascular risk and the risk of heart failure.”
General cardiologist	18	“For example, patients with rheumatoid diseases have an increased risk in the CV areas well, and tissue test of damaged vessels quite clearly show us that we can see that there was inflammation present. We have known for years and years that there is an inflammation reaction, so there can be no doubt that it is indeed a factor.”
Nephrologist	14	“People with CKD develop a micro-inflammatory that increases the renal and cardiovascular risks. This goes crescendo. The more they evolve, the more they will tend to have an inflammation. This can be accentuated by metabolic factors such as diabetes, overweight, that are also pro-inflammatory states.”
GP	10	“Inflammation, for me, there's a systemic inflammatory disease with the AVS, with CRP, with associated symptoms. So generally, I have biological markers that are alert, and often patients have other symptoms when there are systemic and autoimmune diseases, there are often other symptoms than just renal tint, there can be polyarthralgia.”
**Systemic inflammation regardless of underlying cause**		
General cardiologist	12	“There are more and more indications that systemic inflammation in the vessels can cause cholesterol to bind to the walls of the blood vessels.”
Nephrologist	21	“I believe everything works together: smoking, abdominal obesity, lack of exercise, poor diet, all these factors ultimately lead to increased inflammation and this exact inflammation—whether as an intermediate step or directly remains to be seen—is one of the very important building blocks that shows the degree or speed of progression of atherosclerosis”
GP	12	“Heart failure is an inflammatory disease. Heart failure is a systemic process that has an impact on multiple levels and, as a result, it causes inflammation. Patients with heart failure have activation of the inflammation pathways at endogenous level.”
**Target population for systemic inflammation testing**		
General cardiologist	20	“I believe it would be done, if ever, especially in those patients with more events or those with more stenosis to rule out. It would apply to this population group.”
General cardiologist	7	“It depends, but in principle, as soon as the risk factors are known, including family history or smoking, one is more sensitized and starts screening, of course.”
General cardiologist	14	“For patients who have risk factors like diabetes, kidney failure, COPD, autoimmune diseases… Then [systemic inflammation] will be monitored more closely, and we will be more careful.”
Nephrologist	16	“Usually, if I don't suspect specific diseases, I don't evaluate [systemic inflammation] directly, but if I suspect it and the symptoms correlate, then I do a thorough analysis and if it can be systemic inflammation, such as swollen limbs, particular rashes, rheumatic diseases in general, then I refer them.”
General practitioner	22	“In an ideal world, I could request famous acute-phase reactants like C-reactive protein, erythrocyte sedimentation rate, but in a patient who does not initially present with these issues or does not mention any relevant history, routinely checking everyone for inflammation is not common practice unless I suspect there might be an underlying inflammatory or autoimmune condition.”
**Clinical impact of systemic inflammation**		
General cardiologist	11	“We will try to understand where it comes from. If we are talking about minimal inflammation detected with hsCRP, I think we would not do much with it [due to] the lack of treatment and the lack of evidence.”
General cardiologist	20	“I mean, we do not do anything unless there are signs of infection or signs of an inflammatory disease, I do not know, an autoimmune inflammatory disease. But in a person who does not have any of these signs, meaning you do not suspect they have rheumatoid arthritis, ulcerative colitis, or a similar disease, and you do not suspect an infection, you do not do anything with the inflammation.”
**Colchicine treatment in patients with ASCVD + CKD and HF**		
General cardiologist	15	“The main drawback of colchicine is evidence. There is contradiction evidence in that sense. ESC guidelines recommend the use of colchicine but without a strong evidence level. Another drawback is tolerability.”
General cardiologist	20	“[…] colchicine, for example, which we use for pericarditis, has an adverse effect that often limits its use.”

ASCVD, atherosclerotic cardiovascular disease; CKD, chronic kidney disease; CV, cardiovascular; HF, heart failure.

### HCP perceptions of inflammation as a CV risk factor

Two of the interviewees (3% of 62 total HCPs; 1 cardiologist and 1 nephrologist) mentioned systemic inflammation unprompted as a risk factor that they assessed as part of ASCVD + CKD or HF diagnostic or management activities. In both cases, while proactively mentioned, their assessment of inflammation was limited to patients with symptoms of inflammation to determine the underlying cause of the inflammation and to treat it.

Overall, most HCPs, 52 (84% of 62), when asked directly, recognized inflammation as an independent CV risk factor. The level of recognition was highest among nephrologists (92%; *N* = 11 of 12) compared with cardiologists (86%; *N* = 31 of 36) and GPs (71%; *N* = 10 of 14) (Figure 1).

**Figure 1:**
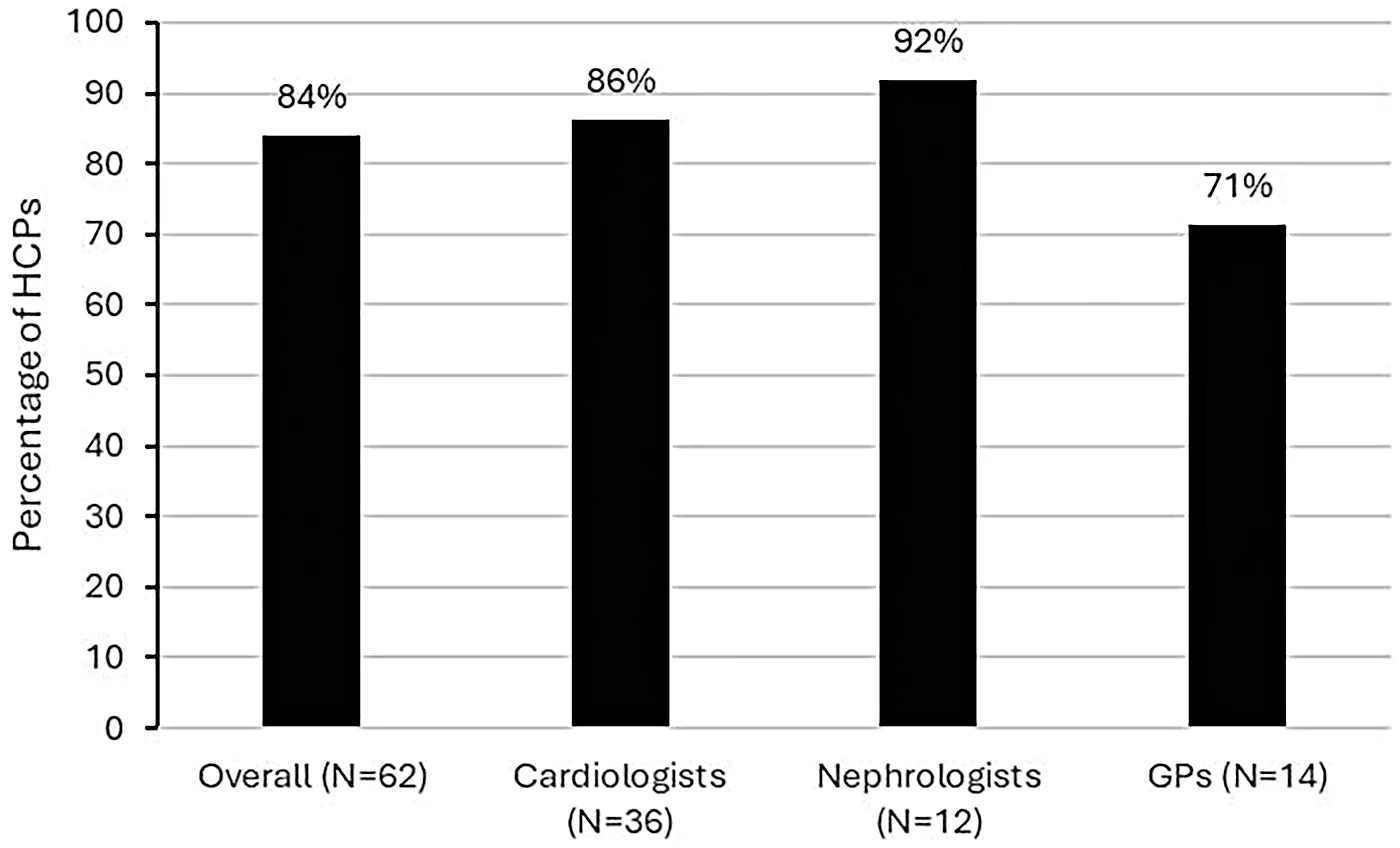
Percentage of HCPs acknowledging inflammation as an independent CV risk factor (*N* = 62). GP, general practitioner; HCP, Healthcare physician.

When discussing inflammation as a residual CV risk factor, some HCPs (6% of 62) specifically referred to inflammation caused by autoimmune diseases, such as psoriatic arthritis (Figure 2). Another 35% of the 62 HCPs discussed inflammation regardless of the underlying cause. Most HCPs in the study (58% of 62 total) discussed inflammation caused by autoimmune disease and other causes, interchangeably.

**Figure 2:**
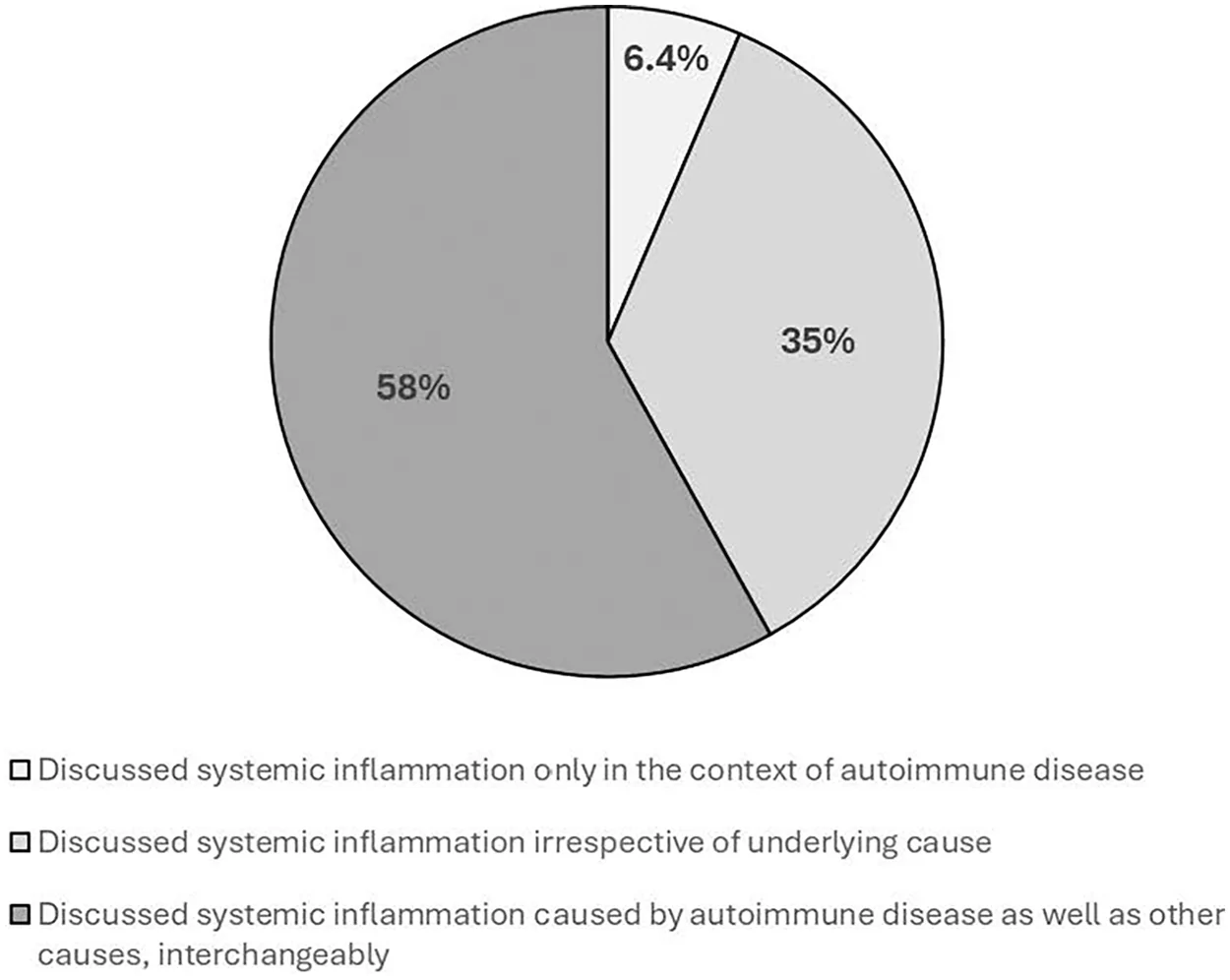
HCPs' understanding of inflammation as a residual CV risk factor (*N* = 62). CV, cardiovascular; HCP, Healthcare physician; SI, systemic inflammation.

The 5 cardiologists (14% of 36) who did not consider inflammation to be an independent CV risk factor indicated that they considered inflammation to be a marker of CV risk, a risk modifier, and a marker of underlying chronic health conditions (e.g., diabetes and kidney failure). In addition, 1 nephrologist (8% of 12) was not aware of any evidence to support inflammation as an independent risk factor for CVD and therefore was not sure whether inflammation is an independent CV risk factor. The 4 GPs (29% of 14) who were not aware of evidence supporting inflammation as an independent CV risk factor were unsure of the role or importance that inflammation plays in CVD.

Twenty of the HCPs who were interviewed for the ASCVD indication (12 cardiologists and 8 GPs) were asked about the perceived importance of inflammation in development and progression of ASCVD (Figure 3A; Supplementary Table S4). Of these HCPs, 7 (35% of 20; 4 cardiologists and 3 GPs) indicated that inflammation is *very important* to development and progression of ASCVD while 11 (55% of 20; 8 cardiologists and 3 GPs) indicated that it is *important*. When discussing the importance of inflammation, HCPs often described the mechanism by which it can affect ASCVD risk yet highlighted that while important in development and progression of ASCVD, inflammation as assessed by hsCRP testing does not have a demonstrated clinical role due to the lack of available effective treatments.

**Figure 3:**
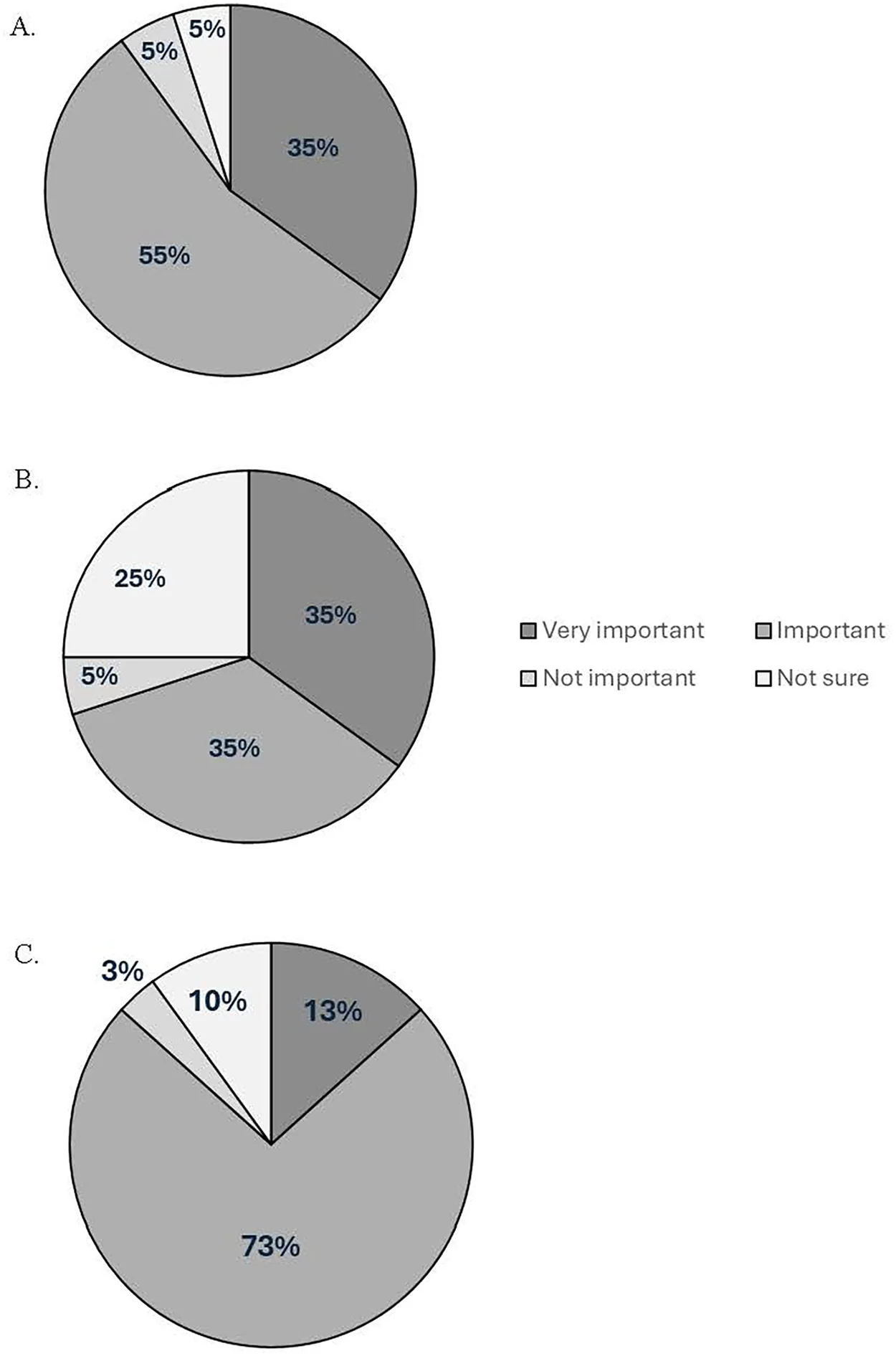
Perceived importance of inflammation in the development and progression of ASCVD, CKD, and HF. (**A**) ASCVD (*N* = 20); % of HCPs. (**B**) CKD (*N* = 20); % of HCPs. (**C**) HF (*N* = 30); % of HCPs.

Twenty of the HCPs who were interviewed for the CKD indication (12 nephrologists and 8 GPs) were asked about the perceived magnitude of importance of inflammation in development and progression of CKD (Figure 3B; Supplementary Table S4). Of these HCPs, 7 (35% of 20; 5 nephrologists and 2 GPs) indicated that inflammation is *very important,* and another 7 (35% of 20; 2 nephrologists and 5 GPs) indicated that it is *important*. Three nephrologists (25% of 12) indicated that the presence of inflammation can lead to an accelerated loss of kidney function.

Thirty HCPs who were interviewed for the HF indication (24 cardiologists and 6 GPs) were asked about the perceived magnitude of importance of inflammation in development and progression of HF (Figure 3C; Supplementary Table S4). Of these HCPs, 4 (13% of 30; 3 cardiologists and 1 GP) indicated that inflammation is *very important* to development and progression of HF, while 22 (73% of 30; 18 cardiologists and 4 GPs) indicated that it is *important*.

### Inflammation testing in current practice: target population and tests used

Overall, 29 of the 62 HCPs (47%) routinely test for inflammation: 16 cardiologists (44% of 36), 8 nephrologists (67% of 12) and 5 GPs (36% of 14). Overall, 40 of the 62 HCPs (65%) performed any testing for inflammation: 14 (35% of 40) did so in up to 25% of their patients, 6 (15% of 40) in 26% to 50% of their patients, 1 (3% of 40) in 51% to 75% of their patients, and 13 (33% of 40) in >75% of their patients. Six of the 40 HCPs (15%) did not provide the exact proportion of patients tested for inflammation.

In many cases, HCPs indicated that inflammation assessment was primarily targeted toward patients with suspected underlying inflammatory conditions, autoimmune conditions, or other CV risk factors (e.g., diabetes, atherosclerosis, high cholesterol, and smoking). Approximately half of the HCPs interviewed did not routinely assess for inflammation (53%; 62) based on the lack of perceived relevance given the lack of availability of effective treatments and the absence of a demonstrated role in clinical decision-making.

Among the 40 HCPs of the 62 who did any testing for inflammation, 17 (43% of 40 who test for inflammation; 27% of all 62 HCPs interviewed) did so using hsCRP testing. Other tests mentioned by HCPs who did not use hsCRP included conventional CRP measurement, erythrocyte sedimentation rate, leukocyte count, and procalcitonin. There was considerable heterogeneity in the use of hsCRP testing according to HCP specialty. Among those testing for hsCRP, 12 were cardiologists (33% of 36 cardiologists), 5 were nephrologists (42% of 12 nephrologists), and none were GPs. After acknowledging the role of inflammation in CVD (47 HCPs: 9 GPs, 30 cardiologists, and 8 nephrologists), the HCPs were asked about the key barriers to hsCRP testing (Figure 4). The lack of evidence for treatment of inflammation was mentioned by 22% of GPs, 57% of cardiologists, and 62% of nephrologists. The lack of availability of hsCRP testing or the lack of reimbursement was mentioned by 33% of GPs, 30% of cardiologists, and 25% of nephrologists. The lack of data on how inflammation affects CVD events was mentioned by 22% of GPs, 27% of cardiologists, and 12% of nephrologists.

**Figure 4:**
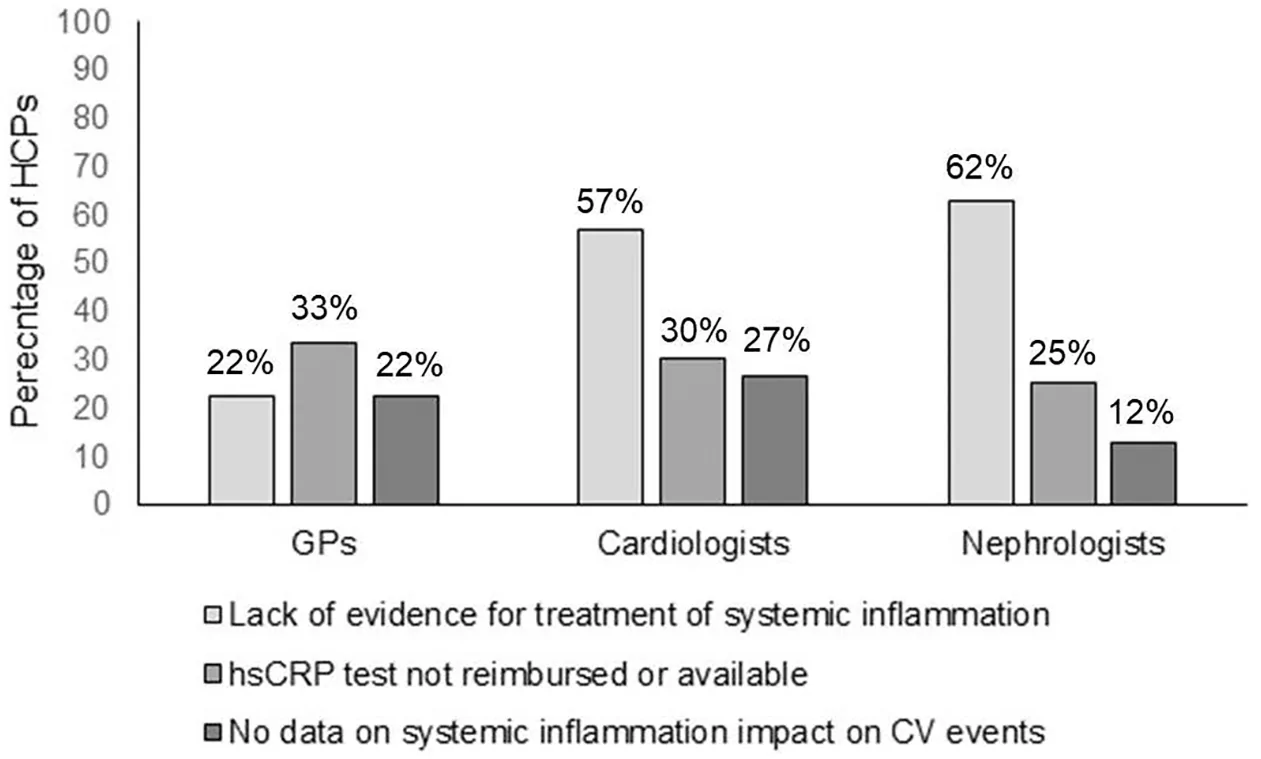
Barriers to hsCRP testing. CV, Cardiovascular; hsCRP, high-sensitivity C-reactive protein. GP, general practitioner.

### Clinical care pathway for patients with ASCVD + CKD

In patients with ASCVD + CKD specifically, there was a limited role of inflammation on initial treatments, ongoing management, rehabilitation, and secondary prevention (Figure 5). However, there were some HCPs who reported managing traditional ASCVD risk factors more aggressively and treating the underlying cause of inflammation in the presence of measured inflammation.

**Figure 5:**
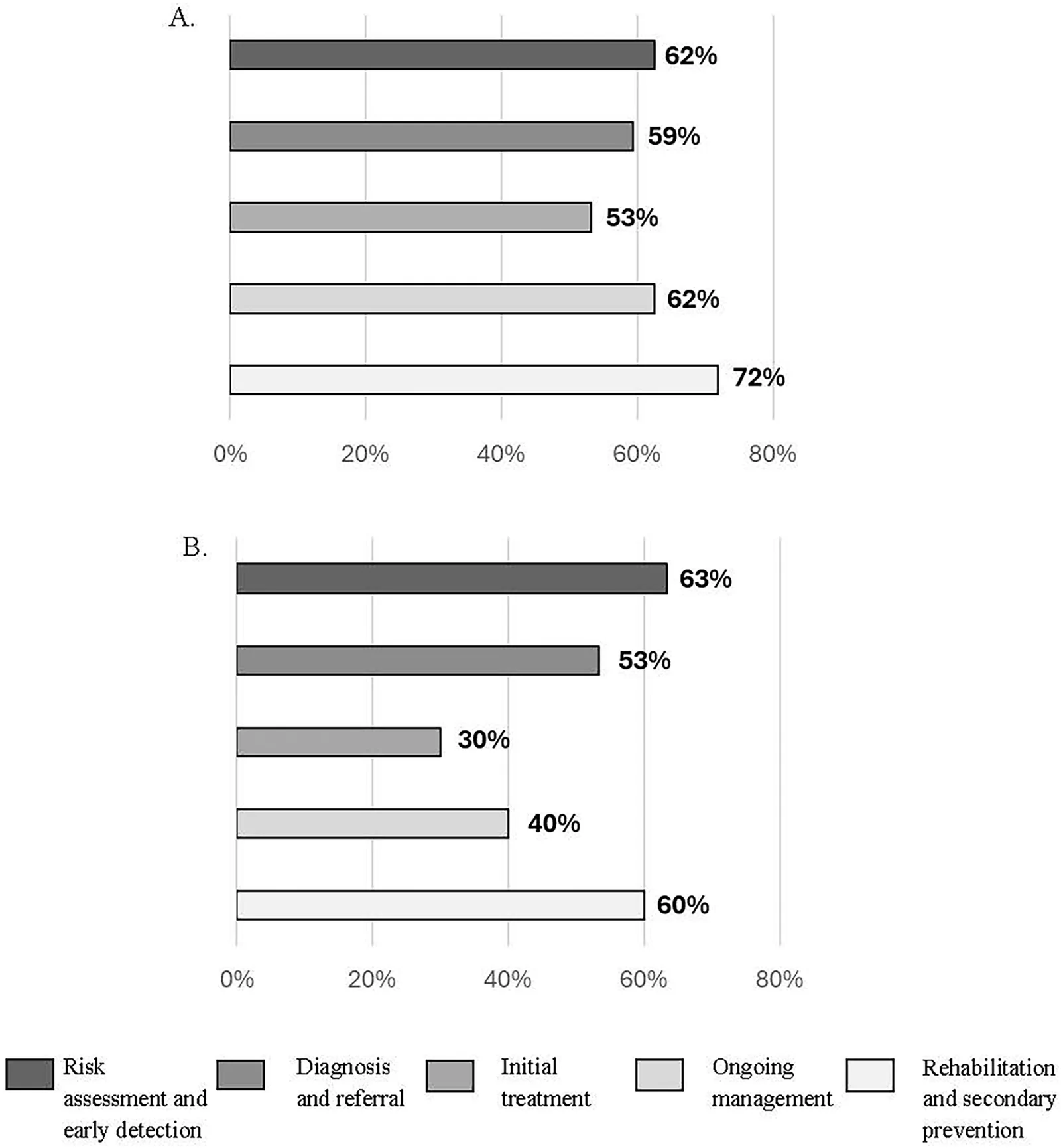
Impact of inflammation on clinical care pathway. ASCVD, Atherosclerotic cardiovascular disease; CKD, Chronic kidney disease; HF, Heart failure. (**A**) ASCVD/CKD. (**B**) HF.

Three (5% of the 62) HCPs mentioned using colchicine as a treatment in patients with ASCVD + CKD unprompted, while 6 (10% of the 62) HCPs (3 cardiologists and 3 nephrologists) acknowledged using it in the treatment of ASCVD + CKD when further prompted. Patients who were treated with colchicine included those with recurrent CVD, those with optimally controlled traditional risk factors, and post-MI patients with elevated hsCRP. HCPs cited a series of barriers to more widespread use of colchicine including poor tolerability, contraindications in stage 4 CKD, polypharmacy, and the limited strength (Class IIb) of the recommendation for colchicine use in ESC guidelines available at the time of the interviews.

### Clinical care pathway for patients with HF

In patients with HF, according to the HCPs, the presence of inflammation did not have a significant role across the clinical pathway unless patients showed signs or symptoms of inflammation or had known underlying inflammatory conditions (Figure 5).

Four HCPs (7% of 62; 3 cardiologists and 1 GP) mentioned colchicine, as a treatment for inflammation in HF unprompted. Cardiologists reported using it occasionally as a treatment in patients with HF. GPs were aware of colchicine treatment, but its use was limited to specialists.

Patients with HF targeted with colchicine treatment by HCPs included those with ischemic heart disease, myocarditis, pericarditis, post-heart valve replacement, and patients experiencing symptom changes accompanied by increased hsCRP. Participating HCPs cited barriers to more widespread use of colchicine in patients with HF such as adverse effects like diarrhea, compatibility with other medications, and other contraindications.

## Discussion

### Key findings

In this qualitative interview-based study conducted across 6 European countries and 3 clinical specialties, the perception and practices of a sample of HCPs treating patients with ASCVD + CKD or HF regarding inflammation were assessed. Participating HCPs treating patients with ASCVD + CKD or HF rarely acknowledged the importance of inflammation in the development or progression of disease, or as a key factor in patient clinical management without being prompted. Only 3% of the 62 HCPs in this study mentioned inflammation unprompted. In contrast, nearly all other known risk factors (e.g., high cholesterol, diabetes, and obesity) were raised spontaneously and almost universally. When participating HCPs discussed inflammation, the concepts of lower-grade inflammation (irrespective of autoimmune disease) and inflammation due to autoimmune disease were often discussed interchangeably, without distinction.

When prompted, participating HCPs widely perceived inflammation to be an important CV risk factor, though some considered it to be a marker of CV risk, a risk modifier, or an indicator of underlying issues rather than an independent risk factor. Despite this broad acknowledgement (even as simply a marker of risk), testing for inflammation was not routine in clinical practice. Even among the HCPs who did consider testing for inflammation as routine, testing was often targeted to patients with exacerbation of symptoms or with underlying inflammatory conditions. Only 13 of 62 HCPs (5 GPs, 6 cardiologists, and 2 nephrologists) indicated that testing for inflammation was routine and broadly performed (i.e., in more than 75% of their patients). Furthermore, among HCPs who tested for inflammation, less than half used hsCRP, while the remaining HCPs used conventional CRP measurement, erythrocyte sedimentation rate, leukocyte count, and procalcitonin tests.

Overall, according to the interviewed HCPs, the assessment of inflammation has limited application in routine clinical practice due to a lack of (up until recently) guideline recommendations and treatment options. In the presence of inflammation, interviewed HCPs indicated that they focused on controlling the underlying conditions and risk factors that can contribute to inflammation. Few HCPs mentioned using colchicine in these patients, citing poor tolerability, contraindications in stage 4 CKD, polypharmacy, and the limited strength of the recommendation in the 2023 ESC Guidelines (Class IIb; i.e., usefulness or efficacy is less well established) (35). In the 2024 ESC chronic coronary syndrome (CCS) Guidelines (published after this study was completed), both measuring hsCRP levels in the initial diagnostic management and considering low-dose colchicine in patients with atherosclerotic coronary artery disease are Class IIa recommendations with the weight of evidence in favor of usefulness/efficacy (38).

### Clinical implications and integration into standard practice

The present study demonstrated that monitoring for inflammation was not routine in clinical practice in patients with ASCVD + CKD or HF among participating HCPs. However, HCPs acknowledged that inflammation plays a role across the clinical pathways and could affect secondary prevention. HCPs suggested three strategies to increase awareness and further implement monitoring of inflammation in clinical practice. Strategies included providing additional evidence of the role of inflammation as an independent risk factor, updating treatment guidelines based on this evidence, and undertaking a robust outreach campaign to increase awareness across geographies and medical specialities about the role of inflammation in patients with ASCVD + CKD or HF.

### Other studies and implications

Data on HCP perceptions regarding the role of inflammation in people with ASCVD + CKD or HF are limited. In the cross-sectional FLAME-ASCVD survey-based study in cardiologists, 71% of 602 eligible cardiologists from 10 countries agreed that inflammation was a risk factor for ASCVD, aligning closely with the findings of the current study (86%) (14, 15). Furthermore, 64% of HCPs indicated in the present study that they tested for inflammation, with 41% of these cardiologists indicating that the results of the tests influenced their decision to initiate anti-inflammatory treatment. Overall, 47% of cardiologists used hsCRP as a test for inflammation, and 45% stated that further guidelines were required to establish testing in clinical practice.

In the FLAME-ASCVD-Nephro survey, nephrologists from across 10 countries who had been practicing for ≥3 years and who had treated ≥20 patients/month with ASCVD + CKD were surveyed about their awareness and perception of systemic inflammation (15). In this survey, nephrologists considered systemic inflammation a CV risk factor in ASCVD + CKD patients. However, they felt that the risk of systemic inflammation was not at the level of traditional CV risk factors. The most common reported unmet need in ASCVD + CKD patients was the lack of effective treatment options for systemic inflammation. Both the FLAME-ASCVD-Nephro survey and the present HCP survey suggest that further efforts are needed to raise HCP awareness of the role of inflammation and the importance of testing hsCRP levels in patients with ASCVD + CKD.

Growing evidence supports the central role of inflammation in the development and progression of ASCVD and the inclusion of hsCRP in the recommended SMART risk calculator for estimating risk of recurrent CV events (45). Despite the 2021 ESC guideline recommendation that low-dose colchicine may be considered in secondary prevention if other risk factors are not sufficiently controlled or if recurrent CVD events occur under optimal therapy (36), only 6 of the HCPs interviewed in the current study used colchicine in the treatment of patients with ASCVD + CKD.

The 2023 ESC Guidelines for the management of acute coronary syndromes identified several key gaps in the evidence base for inflammation, specifically relating to inflammation as a treatment target and the use of biomarkers of inflammation to guide treatment of residual risk (35). Given these identified evidence gaps and the lack of recommendations, it is unsurprising that HCPs rarely considered inflammation in the clinical care pathways of patients with ASCVD + CKD. The present study was conducted before the release of the 2024 ESC Guidelines for management of CCS in which Class IIa recommendations regarding hsCRP testing and low-dose colchicine use have been added (38). With the recent update of the guidelines, it is anticipated that hsCRP testing may become more widespread.

Several recent publications have demonstrated that systemic inflammation contributes to the development and progression of ASCVD including atherosclerosis, HF, MI, and pericarditis (46, 47). Inflammatory processes can cause plaque instability (47) and myocardial damage leading to fibrosis, remodeling and dysfunction (48). hsCRP screening is mentioned as a way to assess risk and identify candidates for anti-inflammatory treatment and imaging modalities are also being used to detect vascular inflammation (46).

### Strengths and limitations

There are several important strengths of this study. This study was conducted across 6 different countries and across 3 clinical specialties, covering the full and diverse responses across the ASCVD + CKD and HF clinical spectrum, from patient risk assessment and diagnosis to long-term patient follow-up. The interviews were 60-minutes long, thereby capturing a wealth of data on the attitudes and practices of HCPs relating to inflammation in the clinical care pathways for patients with ASCVD + CKD or HF.

The study was, however, limited by the number of participants included in the study and the semi-structured interview approach and thus should be considered qualitative in nature rather than quantitative. Additional limitations include the low number of participating HCPs from each country, which may affect the generalizability of the findings. The study is also limited to the specific time frame, with physicians' attitudes and perceptions likely to have evolved with the development of new treatments and treatment guidelines with respect to inflammation. Finally, the relaxation of the GP eligibility threshold from ≥10 to ≥5 ASCVD + CKD patients may have resulted in the inclusion of GPs with more limited experience managing this patient population, potentially influencing the perspectives captured.

## Conclusions

This cross-European, qualitative, interview-based study of HCPs including cardiologists, nephrologists, and GPs was designed to assess their perceptions and practices with respect to the role of inflammation in the treatment of patients across the spectrum of CVD (including ASCVD + CKD and HF). Among participating HCPs inflammation was not routinely assessed and the use of hsCRP testing was limited. Furthermore, when participating HCPs did assess inflammation, it did not frequently alter the patient management approach, leaving this residual risk in secondary prevention largely unaddressed.

The findings of our study likely reflect the limited guideline recommendations and their implementation relating to inflammation and specifically the lack of clear therapeutic pathways for managing inflammation levels, lack of effective treatments, and lack of understanding of the benefits of management of inflammation. All pillars of CV risk assessment and reduction strategies including cholesterol, metabolic, thrombotic, triglyceride, lipoprotein(a), and inflammatory risk should be addressed in secondary prevention on an individually tailored basis.

The burden of ASCVD is enduring and substantial. This is despite the widespread use of therapies to manage traditional CV risk factors such as lipid-lowering agents and blood pressure medications in secondary prevention. Exploiting strategies targeting inflammation may help to reducing residual CV risk. Successful targeting of inflammation in clinical care pathways for patients with ASCVD + CKD or HF will require increasing both the evidence and the awareness of the role of inflammation in CVD.

## Data Availability

The dataset presented in this article is available upon reasonable request to the corresponding author, janhein.cornel@gmail.com.
