# Supplementary material for: Inflammation across the spectrum of cardiovascular disease: healthcare physician perceptions and practices in six European countries
Source: Front Cardiovasc Med. 2026 Sep 25;13:1672079. doi: 10.3389/fcvm.2026.1672079 (PMC13651106; doi:10.3389/fcvm.2026.1672079)
Supplement: Supplementary file 1 [file Datasheet1.pdf]

frontiers

Supplementary Material

Systemic inflammation across the spectrum of cardiovascular disease: Healthcare physician perceptions and practices in six European countries

Guiomar Mendieta[1,2], Morten Schou[3], Jelena Vukmirica[4], Henna Cederberg-Tamminen[4], Edmundo Erazo-Tapia[4], Roman Casciano[5], Stephanie Read[6], Jelena Sostar[7], Marine Mathias[8], April Dominique Ocampo[9], Andrea Bernardini[6], Jan H Cornel[10]*

[1]Department of Cardiology, Hospital de la Santa Creu i Sant Pau, Barcelona, Spain
[2]Centro Nacional de Investigaciones Cardiovasculares (CNIC), Madrid, Spain
[3]Department of Cardiology, Copenhagen University Hospital–Herlev & Gentofte, Denmark
[4]Novo Nordisk A/S, Copenhagen, Denmark
[5]Certara, New York, NY 10017, USA
[6]Certara, London, UK
[7]Certara, Milan, Italy
[8]Certara, Paris, France
[9]Certara, Makati City, Philippines
[10]Department of Cardiology, Radboud University Medical Center, Nijmegen, The Netherlands and Department of Cardiology, Northwest Clinics, Alkmaar, The Netherlands
*** Correspondence:**
Jan H. Cornel
janhein.cornel@gmail.com

Supplementary Tables

Supplementary Table S1. Recruitment Quota

	ACSVD + CKD	HF
**GPs**	**8**	**6**
**Cardiologists**	**12**	**24**
**Nephrologists**	**12**	**-**
**Total**	**32**	**30**

ASCVD, atherosclerotic cardiovascular disease; CKD, chronic kidney disease; GP, general practitioner; HF, heart failure

Supplementary Table S2. Study population

Country	Sent screener	HCPs that responded to screener	Eligible HCPs	Interviewed
**France**	60	19	14	11
**Spain**	50	26	19	11
**Denmark**	228	34	17	10
**Sweden**	151	42	21	10
**The Netherlands**	410	24	12	10
**Switzerland**	191	23	18	10
**Total**	1090	168	101	62

Notes: Eligible HCPs were prioritized to be interviewed based on their speciality to fill the quota from each country, and greater years of practice and number of patients treated.
HCP, health care professional

Supplementary Table S3. Reasons for exclusion of HCPs based on screener questions

	**France (n=5)**	**Spain (n=7)**	**Denmark (n=17)**	**Netherlands (n=12)**	**Sweden (n=21)**	**Switzerland (n=4)**	**Total**
Did not agree to Privacy Policy	1	-	-	-	-	1	2
Did not consent to participate in a telephone interview	-	-	-	1	-	-	1
HCP is a government worker	-	-	11		7		18
Prohibited from participating in industry research by employer	-	-	1	1	-	-	2
Not a primary care provider, general cardiologist, nephrologist, or HF specialist	-	-	3	-	9	1	13
≤5 or ≥30 years post fellowship	1	5	1	6	5	2	20
Saw patients with neither ASCVD nor HF	1	-	-	-	-	-	1
Does not treat CVD patients with concomitant CKD stage 3-4	-	1	-	2	-	-	3
<10 patients seen with ASCVD and CKD stage 3-4	1	1	1	1	-	1	5
<10 patients with CKD stage 3-4 seen over the last 3 months	1	-	-	1	-	-	2

ASCVD, atherosclerotic cardiovascular disease; CKD, chronic kidney disease; CV, cardiovascular; HCP, health care professional; HF, heart failure.

Supplementary Table S4. HCP perception of the level of importance of systemic inflammation in development and/or progression of ASCVD, CDK, or HF

	Cardiologists	Nephrologists	GPs	Total
**ASCVD, n (%)**				
**N**	**12**	**N/A**	**8**	**20**
**Very important**	4 (33.3%)	N/A	3 (37.5%)	7 (35.0%)
**Important**	8 (66.6%)	N/A	3 (37.5%)	11 (55.0%)
**Not important**	-	N/A	1 (12.5%)	1 (5.0%)
**Not sure**	-	N/A	1 (12.5%)	1 (5.0%)
**CKD, n (%)**				
**N**	**N/A**	**12**	**8**	**20**
**Very important**	N/A	5 (41.7%)	2 (25.0%)	7 (35.0%)
**Important**	N/A	2 (16.7%)	5 (62.5%)	7 (35.0%)
**Not important**	N/A	1 (8.3%)	-	1 (5.0%)
**Not sure**	N/A	4 (33.3%)	1 (12.5%)	5 (25.0%)
**HF, n (%)**				
**N**	**24**	**N/A**	**6**	**30**
**Very important**	3 (12.5%)	N/A	1 (16.7%)	4 (13.3%)
**Important**	18 (75.0%)	N/A	4 (66.7%)	22 (73.3%)
**Not important**	1 (4.2%)	N/A	-	1 (3.3%)
**Not sure**	2 (8.3%)	N/A	1 (16.7%)	3 (10.0%)

ASCVD, atherosclerotic cardiovascular disease; CKD, chronic kidney disease; GP, general practitioner; HF, heart failure; N/A, not applicable
